# College Prizes in London

**Published:** 1861-07

**Authors:** 


COLLEGE PRIZES IN LONDON.—The following prize subjects are now offered by the Council of the Royal College of Surgeons of England for competition among the fellows and members of that institution. The subject of the collegial triennial anatomical prize of fifty guineas is "The Anatomy and Physiology of the Supra-Renal Bodies." There are two Jacksonian prizes of twenty guineas for the present year, namely, "The Structure and Diseases of the Lachrymal Passages at the Inner Side of the Orbit, being those between the Conjunctiva and the Nasal Cavity;" the other is "On the Best Method of Effecting the Radical Cure of Inguinal Hernia, explaining the Principle of the Operation adopted." There are also two subjects for the Jacksonian prize for the ensuing year, 1862, namely, "The Relative Value of the Treatment of Popliteal Aneurism by Ligature and by Compression;" and the other, "On the Healthy and Morbid Anatomy of the Tonsils, and the appropriate Treatment of their Diseases." The last Jacksonian prize was delivered at a meeting of the Council on Thursday afternoon, to Mr. Henry Thompson, F.R.C.S., of Wimpole Street.—*London Lancet.*

